# A Critical Study on DNA Probes Attached to Microplate for CRISPR/Cas12 Trans-Cleavage Activity

**DOI:** 10.3390/bios13080824

**Published:** 2023-08-17

**Authors:** Konstantin M. Burkin, Aleksandr V. Ivanov, Anatoly V. Zherdev, Boris B. Dzantiev, Irina V. Safenkova

**Affiliations:** A.N. Bach Institute of Biochemistry, Research Centre of Biotechnology of the Russian Academy of Sciences, 119071 Moscow, Russia; burkin-kost@yandex.ru (K.M.B.); a.ivanov@fbras.ru (A.V.I.); zherdev@inbi.ras.ru (A.V.Z.); dzantiev@inbi.ras.ru (B.B.D.)

**Keywords:** CRISPR-Cas12, trans-cleavage, microplate, DNA trans-target, DNA amplification, isothermal amplification, SARS-CoV-2

## Abstract

CRISPR/Cas12-based biosensors are emerging tools for diagnostics. However, their application of heterogeneous formats needs the efficient detection of Cas12 activity. We investigated DNA probes attached to the microplate surface and cleaved by Cas12a. Single-stranded (ss) DNA probes (19 variants) and combined probes with double-stranded (ds) and ssDNA parts (eight variants) were compared. The cleavage efficiency of dsDNA-probes demonstrated a bell-shaped dependence on their length, with a cleavage maximum of 50%. On the other hand, the cleavage efficiency of ssDNA probes increased monotonously, reaching 70%. The most effective ssDNA probes were integrated with fluorescein, antibodies, and peroxidase conjugates as reporters for fluorescent, lateral flow, and chemiluminescent detection. Long ssDNA probes (120–145 nt) proved the best for detecting Cas12a trans-activity for all of the tested variants. We proposed a test system for the detection of the nucleocapsid (N) gene of SARS-CoV-2 based on Cas12 and the ssDNA-probe attached to the microplate surface; its fluorescent limit of detection was 0.86 nM. Being united with pre-amplification using recombinase polymerase, the system reached a detection limit of 0.01 fM, thus confirming the effectiveness of the chosen ssDNA probe for Cas12-based biosensors.

## 1. Introduction

Biosensors for the detection of DNA/RNA-containing analytes are in demand in various fields including medicine, biosafety, agriculture, and food production. The COVID-19 pandemic has posed a great demand for the large-scale detection of pathogen DNA for health organizations [1,2]. The use of inexpensive and user-friendly methods for DNA/RNA detection with high sensitivity and specificity could significantly reduce the cost of testing and facilitate the finding and isolation of infected individuals. The development of such methods remains a pertinent challenge today.

One of the prospective approaches for DNA detection is the use of the CRISPR (Clustered Regularly Interspaced Short Palindromic Repeats)—Cas12 (CRISPR-associated protein 12) system [3,4,5,6]. In contrast to common amplification techniques, this system provides the specific recognition of DNA under isothermal conditions; in most cases, all reactions proceed at 37 °C. This system comprises the Cas12 endonuclease complexed with guide RNA (gRNA). The holoenzyme Cas12-gRNA randomly binds to a DNA molecule and starts its scanning until the protein recognizes a protospacer adjacent motif (PAM). Then, conformational changes of Cas12 unwind 20 bp dsDNA downstream PAM and gRNA hybridizes with the corresponding strand. In the case of the fully complementary hybridization, an R-loop structure is formed [7,8]. Once the gRNA recognition has occurred, the enzyme sequentially cleaves the target double-stranded (ds)DNA in both strands (cis-cleavage). Furthermore, half of the dsDNA dissociates from the R-loop and one of the active cleavage centers becomes available for the non-specific cleavage of single-stranded (ss)DNA (trans-target) [9,10]. Amplification in the CRISPR-Cas12 system relies on multiple trans-nuclease reactions catalyzed by an activated Cas12–gRNA complex [11,12]. The trans-nuclease activity of Cas12 can be detected using labeled ssDNA probes (trans-targets) [11]. Various methods such as fluorophore–quencher pairs, lateral flow tests (LFT), or direct electrochemical detection of cleaved fragments are used for trans-cleavage detection [13,14,15].

Several strategies have been proposed to enhance the sensitivity of CRISPR–Cas12-based analysis [16]. One such strategy is a heterogeneous analysis format when one end of the ssDNA probe is immobilized on a surface, while the other end contains a reporter. This format has demonstrated a 10–30 times higher sensitivity compared to homogeneous assays [17]. Heterogeneous analysis is usually implemented with the use of ultra-dispersed particles (commonly magnetic ones) or a microplate surface. Thus, heterogeneous formats for CRISPR–Cas12 biosensors of different analytes hold promise. In particular, heterogeneous (solid-state) formats gain additional value through their use in highly sensitive nanopore detection techniques [15,18]. However, the choice of DNA probes for heterogeneous hydrolysis is an urgent task. The comparison of DNA probes with different structures and lengths will indicate probes for the most efficient Cas12 hydrolysis. Thus, for DNA probes attached to magnetic particles, the influence of the carrier-reporter distance on the cleavage efficiency was shown [19,20]. To the best of our knowledge, no other systematic studies of DNA probes for trans-cleavage have been performed.

Comparison of microplates and magnetic particles as carriers shows that the microplate has a number of advantages for proceeding with many simultaneous reactions due to the simple non-laborious addition, incubation, and separation of reactants. Analyses based on microplates and magnetic particles demonstrated close sensitivities [21,22].

**Figure 1:**
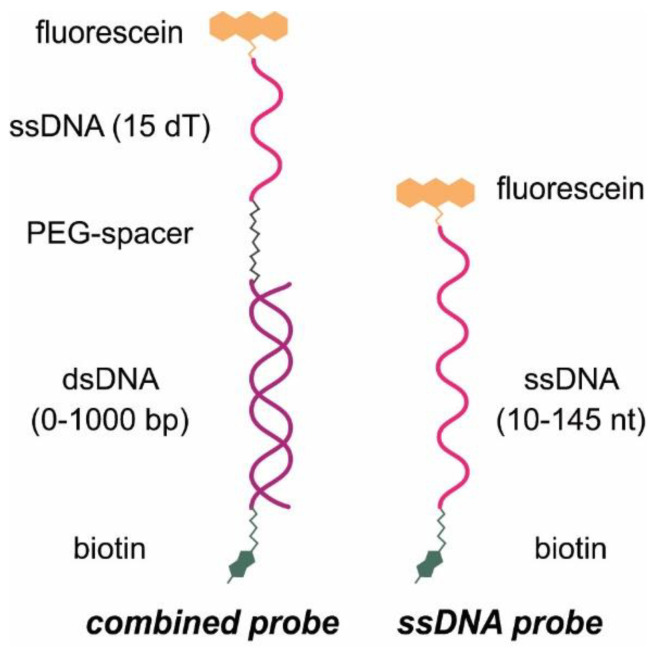
Scheme of the used DNA probes.

In this study, we focused on the heterogeneous CRISPR–Cas12 biosensors to enhance the sensitivity of DNA detection. We compared DNA probes of various structures and lengths attached to a microplate surface and evaluated the efficiency of their hydrolysis by activated Cas12. Two types of DNA probes labeled with fluorescein and biotin were used (scheme of the DNA probes is presented in Figure 1): (1) ssDNA with a length from 10 to 145 nt, and (2) combined probes including dsDNA (0–1000 bp), spacer from ethylene glycol chains, and poly-dT 15 nt. The target DNA was a genome fragment from the bacteria *Dickeya solani*, a pathogen infecting potato crops [23]. Moreover, for ssDNA attached to the microplate surface, different reporters (fluorescein for fluorescence, antibodies for lateral flow test (LFT), and peroxidase conjugates for chemiluminescence) were employed for detection. The most effective DNA probe identified in this study was applied for the detection of the nucleocapsid (N) gene of SARS-CoV-2 to highlight the practical application of our findings.

## 2. Materials and Methods

### 2.1. Materials

Oligonucleotides with modifications (6-fluorescein amidite [FAM], 5-carboxyrhodamine-X [ROX], biotin, Black Hole Quencher-2 [BHQ], and polyethylene glycol [PEG] (C3)) were synthesized by Syntol and Lumiprobe (Moscow, Russia). EnGene LbCas12a, DNAseI, RNAse inhibitor, T7 RNA polymerase, Taq polymerase, Taq buffer, NTPs, a Monarch DNA Gel Extraction Kit, and an RNA cleanup kit were purchased from NEB (Ipswich, MA, USA). The Tersus Polymerase Kit, dNTPs, and unmodified oligonucleotides were obtained from Evrogen (Moscow, Russia). Monoclonal mouse antibodies specific to fluorescein (anti-FAM) were produced by Bialexa (Moscow, Russia); streptavidin was produced by Imtek (Moscow, Russia). Non-target monoclonal mouse antibodies (NT-MAb) were produced by Dr. V.G. Avdienko (Moscow, Russia). Polyclonal goat antibodies against mouse IgG antibodies (GAMI) were obtained from Arista Biologicals (Allentown, PA, USA). An anti-mouse IgG–peroxidase conjugate (anti-mouse-HRP) was produced by Jackson Immuno Research Labs (West Grove, PA, USA). Agarose was produced by Helicon (Lonza Rockland, Rockland, ME, USA). Bovine serum albumin (BSA), casein, sucrose, dithiothreitol, streptavidin-peroxidase polymer (polySTV-HRP) and tetrachloroauric(III) acid hydrate (HAuCl_4_) were purchased from Sigma-Aldrich (St. Louis, MO, USA). The membranes for lateral flow strips were produced by Advanced Microdevices (Ambala Cantt, India) and Sartorius (Göttingen, Germany). Plasmid pGEM-ISG, which contains the ribosomal intergenic spacer (IGS), 596 bp, from *D. solani* was obtained in previous research [24]. All of the used salts and organic compounds had an analytical grade purity.

### 2.2. Synthesis of dsDNA-Probes for Trans-Cleavage

The combined probes including dsDNA with different lengths (40, 80, 120, 160, 300, 500, and 1000 bp) and the same ss-15-dT were obtained by PCR, as described in [19]. Briefly, the reaction mix contained Taq buffer, 167 μM of dNTPs, 200 nM of forward and reverse primers, 1.25 ng/μL of eGFP plasmid, and 0.1 U/μL of Taq polymerase. All of the used primers are presented in Table S1, Supplementary Materials). The PCR was performed by initial annealing at 95 °C for 5 min, 40 cycles of amplification, and the last 5 min of incubation at 72 °C. Each cycle consisted of 30 s denaturation at 95 °C, 30 s primers annealing at 65 °C, and 60 s elongation at 72 °C. The product was concentrated via centrifugation in an Amicon Ultra 3K device (Merck Millipore), purified by electrophoresis in 2% agarose gel, and then extracted by a Monarch DNA Gel Extraction Kit. The concentration of the dsDNA-probes was estimated using a NanoDrop ND-2000 spectrophotometer (Thermo Fischer Scientific, Waltham, MA, USA).

The ds-DNA-probe with 20 bp of the ds-part was obtained by annealing complementary oligonucleotides–eGFP-R-20-Bio with eGFP-F-C_3_PEG-dT15-FAM (see in Table S1, Supplementary Materials): 10 μM of each oligonucleotide was incubated at 80 °C for 2 min, then gradually cooled for 15 min until reaching 20 °C. The probe without the ds-part (FAM-eGFP-R-0-Bio) was used without modifications.

### 2.3. Synthesis of Cis-Target DNA for Recognition by Cas12–gRNA

Two cis-targets for recognizing gRNA–Cas12a were used: a ribosomal intergenic spacer (IGS) from *D. solani* and a nucleocapsid (N) gene fragment from SARS-CoV-2 (sequences are presented in Section S2, Supplementary Materials). The dsDNA fragments (596 bp of IGS, 254 bp of N-gene) were amplified by PCR according to the protocol described in [24]. Briefly, the reaction mix contained Tersus buffer, 200 μM of dNTPs, 200 nM of forward and reverse primers (M13 F and R or N-gene F and R, Table S1, Supplementary Materials), 2 ng/μL of pGEM-IGS plasmid or pAL2T-N2g-9 plasmid, and Tersus polymerase. The PCR, purification, and characterization of the cis-target DNA were performed as described in Section 2.2.

### 2.4. Synthesis and Purity Verification of gRNA

For SARS-CoV-2 recognition, gRNA was synthesized by Syntol (Moscow, Russia), according to Ramachandran et al. [25]. For *D. solani*, gRNA was previously proposed by Ivanov et al. [19]. This gRNA was obtained via in vitro transcription. Briefly, complementary oligonucleotides of the gDNA template containing the T7 promoter followed by 44 bp of the gRNA gene were denatured at 95 °C for 2 min, then annealed by gradually decreasing their temperature to 37 °C for 15 min. The reaction mix contained 40 mM of Tris-HCl, pH of 8.0, 10 mM of DTT, 1.25 mM of NTPs, 2 U/μL of RNAse inhibitor, 7.5 U/μL of T7 RNA polymerase, and 2 μM of the DNA template. The reaction was performed at 37 °C for 3 h, then DNAse I buffer x10 and DNAse I (final concentration of 0.1 U/mL) were added and incubated for 30 min at 37 °C. The obtained gRNA was purified by an RNA Cleanup Kit (NEB, Ipswich, MA, USA). The treatment by the DNAse was repeated twice to remove residuals of the gDNA template. The integrity of the gRNA was checked via electrophoresis in 2% agarose gel. The purity of the gRNAs was evaluated by incubating gRNA with 100 nM Cas12 in Neb2.1 buffer for 10 min with the subsequent addition of the ROX and BHQ labeled poly-dT sequence of 15 nt to a final concentration of 500 nM. The kinetics of the ROX fluorescence was detected by Light Cycler 96 (Roche, Rotkreuz, Switzerland) at an excitation wavelength of 578 nm and an emission wavelength of 604 nm.

### 2.5. Preparation of Reagents for Lateral Flow Test Strips and Test Strips Assembling

To detect antibodies as a released reporter of the cleaved ssDNA probe, lateral flow test strips were used as described in [19]. For this, gold nanoparticles (GNPs) were synthesized, a conjugate of GNPs with GAMI was obtained, and test strips were obtained (test zone: GAMI; control zone: NT-Mab; conjugate: GNP–GAMI conjugate). Detailed methods are presented in Section S2, Supplementary Materials.

### 2.6. Cas12 Activation and Pre-Incubation for Trans-Cleavage

A Cas12 activation and trans-cleavage assay was performed according to the manufacturer’s manual: (NEB, Ipswich, MA, USA) NEB2.1 buffer with 30 nM of gRNA and 30 nM of EnGene LbCas12a was mixed and incubated at 25 °C for 10 min. Furthermore, Cas12 was activated by the addition of the cis-target DNA to the final concentration of 1 nM (“+Cis”) (for the “–Cis” control reaction, the cis-target was not added) and then incubated in a tube for 30 min at 37 °C. Then, 50 μL of the mix was added into the microplate wells with the pre-adsorbed DNA probe.

### 2.7. Trans-Cleavage Assay of DNA Probes in Microplates

The wells of a black, 96-well microplate (Fluoro Nunc MaxiSorp, Thermo Fisher Scientific) were covered with 50 μL of 6 μg/mL of anti-FAM antibody or streptavidin in 50 mM phosphate-buffered saline, pH 7.4, 100 mM NaCl (PBS) at 4 °C overnight. The microplate was washed four times with 300 μL per well of PBS containing 0.05% Triton X-100 (PBST) using a Wellwash automatic washer (Thermo Fisher Scientific, Waltham, MA, USA). Then, 50 μL of the 50 μM DNA probes was added to the wells and incubated for 1 h at 37 °C. The DNA probes that did not bind to the surface (unbound) were taken and their fluorescence was measured at the fluorescein wavelength (extinction 498 nm, emission 517 nm). After washing, the wells were blocked with a casein solution (1 mg/mL) in PBST for 1 h at 37 °C. The excess casein was removed and the wells were washed. The following steps differed for each variant of the assay.

The first variant of the assay implied the direct addition of the activated Cas12 solution to the wells and incubation for 30 min at 37 °C. After cleavage, 50 μL of the solution with the released fluorescein-containing fragments was transferred to the separate wells and mixed with 50 μL of 25 mM Tris-HCl buffer, pH 9.0. After this, the fluorescence was measured using an EnSpire Multipurpose plate reader (PerkinElmer, Waltham, MA, USA) (extinction 498 nm, emission 517 nm). The ratio of the released fluorescein residues was calculated:

$$released,\;\%=\frac{released}{initial-unbound}*100\%.$$

The second variant of the assay implied incubation with anti-FAM antibodies for 1 h at 37 °C; activated Cas12 was then added and incubated for 1 h at 37 °C. After cleavage, 45 μL of the released anti-FAM antibodies were transferred to the separate wells and mixed with 45 μL of 2x PBST. Subsequently, the anti-FAM antibodies were detected using the test strips (see Section 2.5).

The third variant of the assay implied incubation with anti-FAM for 1 h at 37 °C, followed by another incubation with anti-mouse-HRP conjugate for 1 h at 37 °C, then the addition of activated Cas12 and incubation for 1 h at 37 °C. After cleavage, 45 μL of the released anti-FAM-anti-mouse-HRP complex was transferred to the separate wells and mixed with 100 μL of the SuperSignal ELISA Pico Chemiluminescent Substrate (Thermo Scientific, Waltham, MA, USA). The chemiluminescent signals were measured by Zenyth 3100 (Anthos Labtec Instruments, Wals, Austria) for 13 min.

The fourth variant of the assay implied incubation with the polySTV-HRP conjugate followed by the addition of activated Cas12 and incubation for 1 h at 37 °C. In this case, the microplates were initially covered with anti-FAM antibodies so that after assembly with the DNA-probe, the biotin was available for interaction with the polySTV-HRP conjugate. The following manipulations with the released polySTV-HRP conjugate were the same as in the third variant.

Cleavage of the DNA probes with reporter macromolecules was carried out with higher concentrations of gRNA (100 nM), Cas12 (100 nM), and target DNA (3 nM).

### 2.8. Amplification of Cis-Target DNA with Recombinase Polymerase Amplification (RPA)

An RPA was performed using the basic RPA kit (TwistDx, Cambridge, UK). Briefly, 45.5 µL of a solution containing 0.33 µM forward and reverse primers in rehydration buffer was added to the lyophilized pellet from the basic RPA kit. Then, 2 μL of the target DNA and 2.5 μL of 280 mM magnesium acetate were added. The solution was incubated at 39 °C for 20 min. Then, 2 µL of the mixture (final volume was equaled 50 µL) was used as a cis-target of Cas12a for the trans-cleavage assay of DNA-probes in microplates.

### 2.9. Software

Data were processed and visualized by GraphPad Prism 8.0.1 (GraphPad Software, Boston, MA, USA). The color intensity of the LFT strip scans was evaluated using TotalLab TL120 (Nonlinear Dynamics, Newcastle upon Tyne, UK). An integrated value of coloration lower than two arbitrary units (arb.u.) being below the eye-visible level and were set as negative. The design of the gRNAs was performed using CHOPCHOP version 3 (https://chopchop.cbu.uib.no/, accessed on 16 August 2023) [26]. ANOVA and *t*-tests were performed for the comparison of different variants of fluorescent and LFT-based experiments (Supplementary Materials Section S2).

## 3. Results and Discussion

### 3.1. Design of the Proposed Heterogeneous Assay Based on CRISPR/Cas12 and Attached DNA Probes

**Figure 2:**
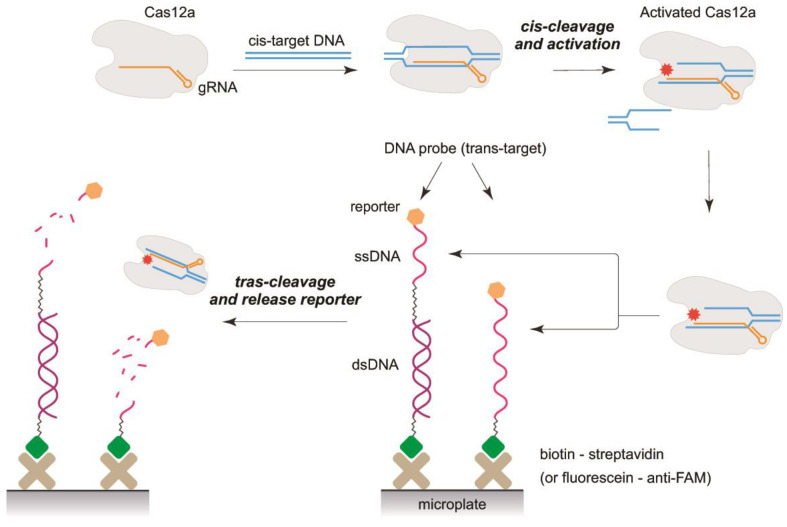
The scheme of the assays.

To detect the trans-nuclease activity of Cas12a in a heterogeneous format, the following scheme was proposed (Figure 2). The Cas12–gRNA complex was assembled and recognized the added cis-target dsDNA. After this, the complex with the activated Cas12a was added to the microplate with attached DNA probes and acquired the trans-nuclease activity that is accomplished with the release of the labeled ssDNA-part of the probes into the solution. If the cis-target DNA was absent, the release of the label did not occur.

Presumably, Cas12a has steric hindrance in the interaction with surface-attached DNA probes. This hindrance limits the rate and efficiency of the hydrolysis. For longer ssDNA probes, increasing the efficiency of DNA probe cleavage and the release of the reporter into the solution are expected. Long ssDNA probes can potentially reduce steric limitations for Cas12a acting near the microplate surface. However, the influence of the ssDNA length could have additional features due to the flexibility of the ssDNA chain and its ability to form additional structures that prevent hydrolytic contacts. Therefore, two types of DNA probes were considered in our comparative study. The probes of the first type consisted of a dsDNA portion of varied length and a ss poly-dT DNA (15 nt). The addition of the ds-part to the probes increases the distance between the ss-part and the surface to facilitate the hydrolysis. The chosen design of these probes is based on the fact that the persistent length of dsDNA is two-fold higher than the persistent length of ssDNA [27,28]. The probes of the second type were ssDNA (poly-dT, poly-dC, and poly-dA) of variable lengths.

All probes were labeled with biotin and fluorescein at their 5′- and 3′-ends and were immobilized on the microplate surface through biotin–streptavidin or fluorescein–anti-FAM interactions. The opposite end of the DNA probe, which was not involved in immobilization interactions, contained the reporter in native form or modified by the attached biomolecule. Four reporters were used: (1) fluorescein, (2) fluorescein−anti-FAM, (3) fluorescein−anti-FAM−anti-mouse-HRP, and (4) biotin−polySTV-HRP. The addition of the activated Cas12 led to the hydrolytic release of the reporters into the solution. The solutions with the released compounds were transferred into new microplate wells, and the reporters were detected in fluorescent, chemiluminescent, or LFT modes (see Section 2.7).

### 3.2. Optimization of Conditions for Heterogeneous CRISPR/Cas12 Trans-Cleavage

**Figure 3:**
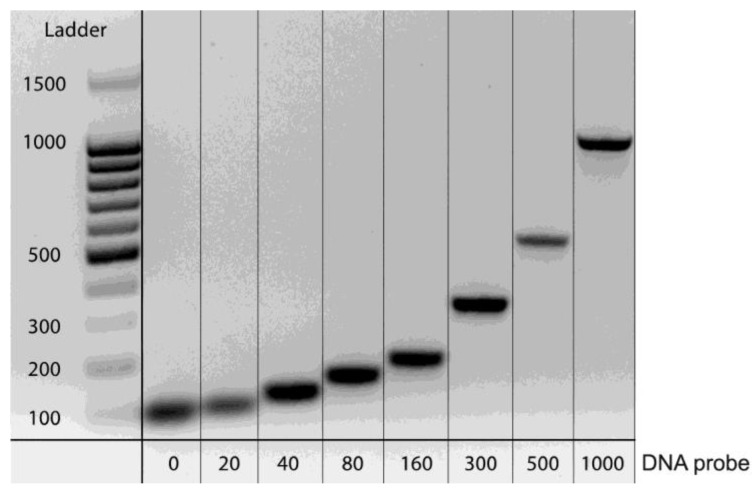
Electrophoresis of the obtained dsDNA probes (with the length of ds part: 0, 20, 40, 80, 160, 300, 1000 nt).

First of all, we obtained all of the necessary components: (1) trans-target DNA probes, (2) cis-target dsDNA, and (3) gRNA. The proposed ssDNA probes were custom synthesized and purified. To obtain combined dsDNA probes, PCR-based synthesis was implemented using primers and a plasmid with a fragment of a green fluorescent protein gene. All dsDNA probes were purified and characterized by electrophoresis. Figure 3 shows that all of the dsDNA probes were mono-products and their lengths corresponded to the expected values.

We obtained two cis-targets: IGS from *D. solani* (for optimizations and comparison of DNA probes in the trans-nuclease reaction with Cas12a) and the N-gene from SARS-CoV-2 (for creation of a heterogeneous assay based on Cas12a). It was found that DNA cis-targets were also mono-products with the expected lengths (Figure S4, Supplementary Materials).

For the recognition of these cis-targets, gRNAs were obtained by in vitro transcription (gRNA for IGS, see its electrophoresis in Figure S5, Supplementary Materials) or were custom synthesized (gRNA for N-gene). Both gRNAs demonstrated the absence of DNA inclusions that could act as cis-target DNAs. The absence of DNA inclusions was confirmed by the fluorescence curves of a homogeneous trans-cleavage of the ROX-dT(15)-BHQ2 DNA probe (Figure S6, Supplementary Materials). Thus, all the necessary DNA and RNA components were obtained and their lengths and purities were confirmed.

**Figure 4:**
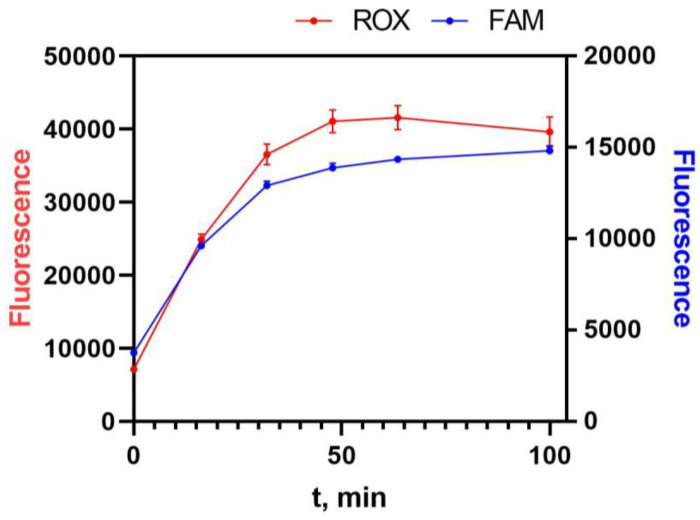
Kinetics of heterogeneous cleavage of biotin-dsDNA-PEG-BHQ-ssDNA-FAM and homogeneous cleavage of ROX-dT(15)-BHQ with activated Cas12a.

Before comparing the efficiency of the DNA probes, the conditions for their sorption and hydrolysis were optimized. Firstly, the hydrolysis time of the DNA probes was studied. The choice of this parameter was based on kinetic experiments performed in parallel for the heterogeneous and homogeneous formats. To register heterogeneous cleavage, combined probes labeled with biotin (on the dsDNA-part) and with FAM and BHQ1 (at opposite ends of the ssDNA-part of the probe) were used. Cleavage of the DNA probe was detected in real-time by recording the fluorescence of the released fluorescein residues. The ROX-dT(15)-BHQ2 probe was added to the reaction volume to record homogeneous hydrolysis. As can be seen in Figure 4, the difference between the heterogeneous and homogeneous formats was insignificant. For the heterogeneous format, 30 min was chosen as the optimal cleavage time. It should be noted that in the case of the macromolecular reporter, the cleavage requires more time due to the steric hindrance for contacts of the activated Cas12a with the cleavage site. Based on our earlier study [29], the cleavage time for macromolecular reporters was chosen to be equal to 60 min.

**Figure 5:**
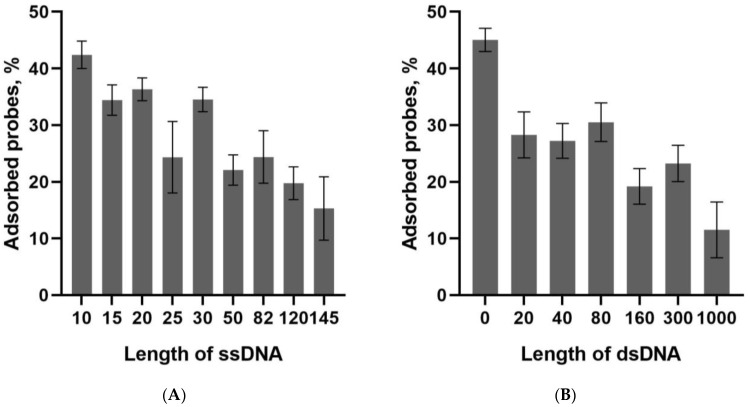
The percentage of ssDNA (**A**) and dsDNA (**B**) probes of different lengths adsorbed after incubation with the streptavidin-coated microplate. The probes were used at a 50 nM concentration.

Secondly, the interaction of biotinylated DNA probes with the microplate surface coated with streptavidin was studied. The concentration of the DNA probes for immobilization was chosen to be 50 nM. Our previous study [29] showed that this concentration provided the maximum coverage for 20 bp dsDNA probes immobilized on the streptavidin-coated microplate. In this study, the 50 nM concentration also provided the maximum coverage for other DNA probes and the probes’ immobilization via the fluorescein–anti-FAM antibody interaction. Figure 5 shows that under the chosen condition, the percentage of the DNA probes binding to the streptavidin-coated microplate ranged from 10% to 45%. Figure S7 in the Supplementary Materials showed similar results for anti-FAM coated microplate. Thus, the increasing length corresponds to a decrease in the binding efficiency (see also Supplementary Materials Section S2 with the results of the one-way ANOVA for a comparison among probes with different lengths). The maximum percentage of bound probes reached 45% for the shortest probes (dsDNA probe 0 bp and ssDNA probe 10 nt), and the minimal value was 10% for the longest probe (dsDNA probe 1000 bp).

Thirdly, the effect of blocking proteins on the hydrolysis of DNA probes was studied. For ssDNA probes of 10–82 nt and dsDNA probes of 20–300 bp, several blocking proteins (casein, BSA, ovalbumin) were compared by their addition after the probes’ adsorption. The compared blocking proteins did not demonstrate reliable differences in the cleavage values for the DNA probes (Figure S8, Supplementary Materials). Thus, any of the compared proteins can be used to prevent non-specific binding.

Thus, the optimal conditions for the cleavage of DNA probes with Cas12a were chosen. DNA probes were adsorbed on the microplate surface from the 50 nM solutions. The surface of the microplate was blocked with casein. Time of Cas12 activation by target DNA: 30 min. Time of hydrolysis of DNA probes: 30 min.

### 3.3. Comparison of DNA Probes in the Trans-Nuclease Reaction with Cas12a

The obtained dsDNA probes (with ds-part lengths 0, 20, 40, 80, 160, 300, and 1000 bp) and ssDNA probes (with poly-dT fragment lengths 10, 15, 20, 25, 30, 50, 82, 120, and 145 nt as well as poly-dA and poly-dC oof 10, 20, 30, 40, and 80 nt) were used to select the most efficient DNA probes for heterogeneous Cas12 hydrolysis.

**Figure 6:**
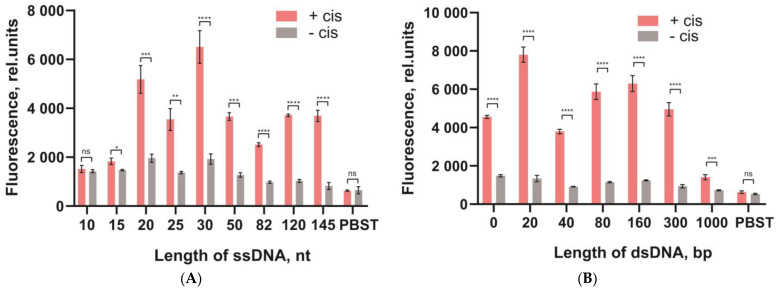

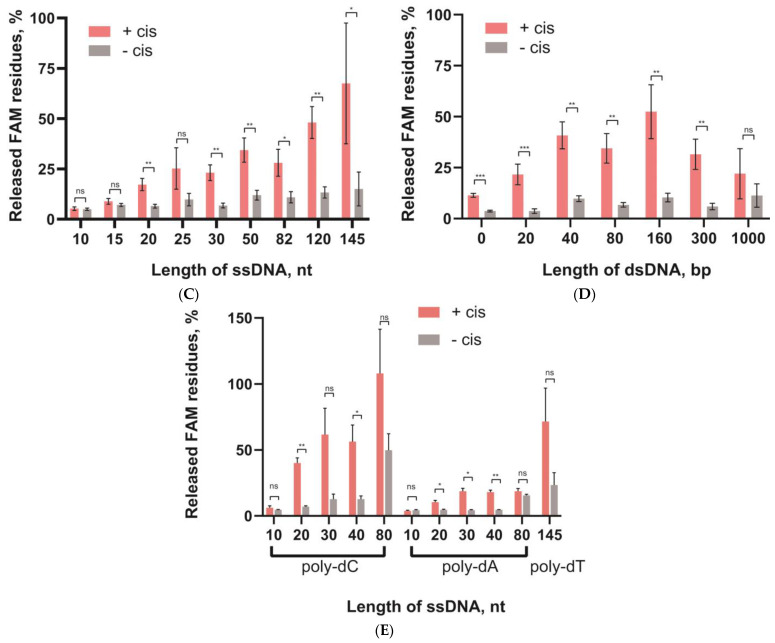
Comparison of the DNA probes attached to the microplate surface and cleaved by Cas12a. Fluorescence without normalization for the ssDNA probes (**A**) and dsDNA probes (**B**) and the percentage (%) of fluorescein released after the cleavage of ssDNA for the poly-dT (**C**), dsDNA probes (**D**), and poly-dC, poly-dT, poly-dA (**E**). Red columns correspond to Cas12a activated by cis-target DNA, and grey columns to the background responses in the absence of cis-target DNA. The significance of the cis-target addition effect for each probe was estimated by the *t*-test. *p*-values of the differences denoted by: ns (*p* > 0.05), * (*p* ≤ 0.05), ** (*p* ≤ 0.01), *** (*p* ≤ 0.001), **** (*p* ≤ 0.0001).

All DNA probes were compared using fluorescein as a reporter. Since different DNA probes bound to the microplate surface in an uneven proportion (see Figure 5), the fluorescence intensity of the released fluorescein residues after cleavage was normalized to the number of bound DNA probes (see Section 2.7). The comparison of DNA probes was performed by measuring the fluorescence of the released fluorescein for two cases: when Cas12 was activated by the cis-target DNA (“+cis”) and in the absence of the cis-target DNA (“−cis”) (background response). Figure 6 shows the fluorescence (A,B) and the percentage of released fluorescein residues (C–E). For the dsDNA probes, the percentage of fluorescein residues released had a bell-shaped dependence on the length of the probes. The highest percentage was 52% for the probe with 160 bp in the ds-part (Figure 6D). For the ssDNA probes (poly-dT), the cleavage was more efficient for longer probes; the highest percentage of cleaved probes was equal to 70% for the longest ssDNA probe (145 nt) (Figure 6C). The two-way ANOVA with Tukey’s correction for two parameters (+cis and −cis) completely proved these conclusions (see the ANOVA results in Supplementary Materials Section S2).

Wang et al. [30] demonstrated that poly-dC probes were more efficient in homogeneous hydrolysis compared to the poly-dA, poly-dT, and poly-dG probes. We also tested the use of poly-dA and poly-dC DNA probes (see Figure 6E). The Poly-dC DNA probes had a high efficiency compared to the same-length poly-dA. The cleavage efficiency of the long poly-dC DNA probes was comparable to that of the 145 nt poly-dT DNA probe.

Since the maximal level of the cleavage efficiency was higher for the ssDNA probes, they were chosen for further studies. Moreover, these probes are much easier to obtain by simple chemical synthesis. Presumably, the use of longer ssDNA probes can increase the efficiency of heterogeneous cleavage.

### 3.4. Comparative Characterization of Signal Marks of Different Types in the Trans-Nuclease Reaction with Cas12

The next stage of our study was to test the integration of the heterogeneous DNA probes with different reporters to provide the possibility of different detection modes: visual (for lateral flow test strips), chemiluminescent, or colorimetric (for enzymatic labels such as peroxidase).

**Figure 7:**
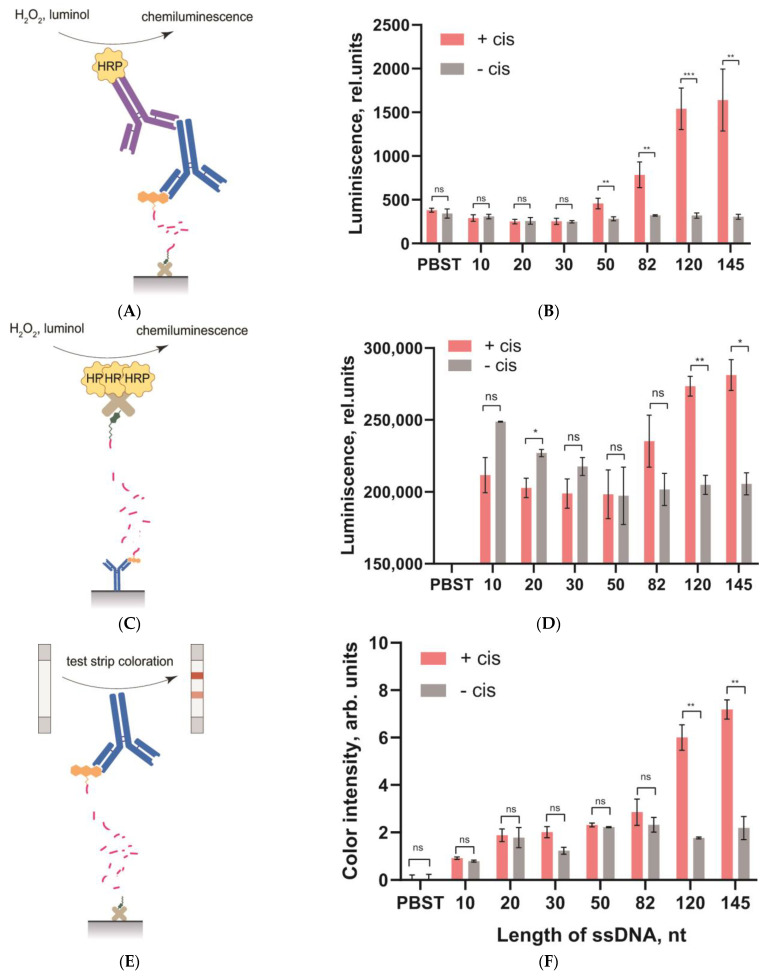
Comparison of the ssDNA probes of different lengths using the anti-FAM–anti-mouse–HRP complex (**A**,**B**), polySTV-HRP (**C**,**D**), and anti-FAM (**E**,**F**) as reporters. The anti-FAM–anti-mouse–HRP complex (**B**) and polySTV-HRP (**D**) released after trans-cleavage were registered by chemiluminescence of the oxidized HRP substrate. The released anti-FAM was registered by the coloration of the test zones of the lateral flow test strips (**E**). The significance of the cis-target addition effect for each probe was estimated by the *t*-test. *p*-values of the differences are denoted by: ns (*p* > 0.05), * (*p* ≤ 0.05), ** (*p* ≤ 0.01), *** (*p* ≤ 0.001).

Thus, three reporters were used: (1) fluorescein–anti-FAM, (2) fluorescein–anti-FAM–anti-mouse–HRP complex, and (3) biotin–polySTV-HRP (Figure 7A,C,E). Both HRP-based assays were performed with a chemiluminescent reaction. Figure 7B,D shows the chemiluminescence of the released HRP-containing complexes after cleavage of the ssDNA probes. As can be seen, the cleavage efficiency for the ssDNA probes also depended on their length, as in Section 3.3. However, the signals for the ssDNA probes with a length of 10–50 nt were practically the same as the background values (see Figure 7B,D). The two-way ANOVA with Tukey’s correction for two parameters (+cis and −cis) proved these conclusions (see the ANOVA results in Supplementary Materials Section S2). Presumably, the cleavage of the DNA probes was suppressed by the presence of large molecular complexes on their top, especially for short-length probes with limited accessibility for Cas12a. The cleavages of the long DNA probes (120 and 145 nt) were effective; the highest signal corresponded to the longest ssDNA probe (145 nt). A large difference was observed for luminescence signals in Figure 7B,D, which was associated with the cross-linking of several HRP molecules in one polySTV-HRP conjugate. Unfortunately, the background signals also increased dramatically for this reporter.

To study the third approach, a series of test strips were made to detect the release of anti-FAM molecules (all of the details with regard to obtaining the LFT strips are presented in Section S3, Supplementary Materials). The LFT for anti-FAM detection was implemented in a sandwich format. When the strip was immersed in a liquid with anti-FAM, a triple complex with the label (GAMI–anti-FAM–GNP-GAMI) was formed in the test zone and coloration appeared. In the absence of anti-FAM, no ternary complex was formed and no coloration was observed. In the control zone, coloration always appeared for both cases due to the formation of a complex (GNP-GAMI–NT-MAb). The obtained LFT strips detected anti-FAM in the range from 5 to 500 ng/mL. Visual detection of coloration corresponded to two or more relative units of color intensity obtained after digital processing of the test strips. The results of cleavage showed that for the short ssDNA probes (10–82 nt), the cleavage efficiency practically did not differ from the background values (Figure 7F). Although there should be less steric limitations for anti-FAM compared to anti-FAM–anti-mouse–HRP and polySTV-HRP, the cleavage efficiency of short probes was not improved. However, the use of long ssDNA probes with a length of 120–145 nt was quite effective.

Therefore, all of the tested macromolecular reporters impeded the cleavage of DNA probes. This fact excludes the use of short probes to detect Cas12a trans-activity in the microplate format. However, probes with a length of 120 and 145 nt provided efficient cleavage for all of the studied cases (these conclusions were confirmed by the ANOVA results, see Supplementary Materials Section S2).

### 3.5. Application of Heterogeneous CRISPR/Cas12 Trans-Cleavage of ssDNA Probe for the Detection of the N-Gene of SARS-CoV-2

To demonstrate the efficiency of the 145 nt ssDNA probe in a heterogeneous assay with Cas12a, we proposed a test system for the detection of the N-gene of SARS-CoV-2 based on Cas12a and the ss-poly-dT-DNA-probe attached to the microplate surface.

The chosen ssDNA probe (poly-dT, 145 nt) with a fluorescent label attached to the streptavidin-coated microplate was used. The Cas12–gRNA complex was assembled and then activated. Samples containing the cis-target DNA (SARS-CoV-2 N-gene fragment) at a concentration of 0.05–5 nM were added to the Cas12–gRNA complex and incubated for 30 min at 37 °C. The mixture was added to the microplate with immobilized DNA probes and incubated for 30 min at 37 °C. The supernatant with the released fluorescein was transferred to other microplate wells and the fluorescence was measured.

**Figure 8:**
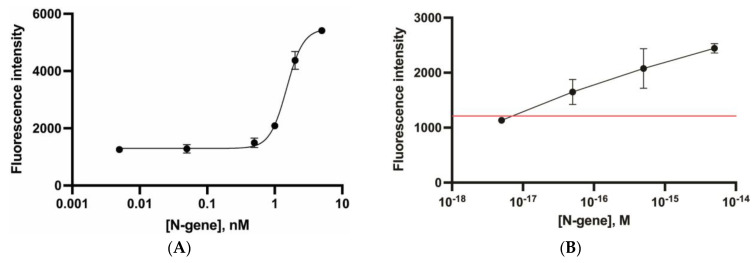
Concentration dependences for the heterogeneous CRISPR/Cas12 trans-cleavage of the ssDNA probe for the detection of the N-gene of SARS-CoV-2 (poly-dT, 145 nt, fluorescein reporter) without cis-target DNA pre-amplification (sigmoid function with parameters: bottom = 1296, top = 5480, IC50 = 1.499, HillSlope = 3.485) (**A**) and with pre-amplification with RPA (linear function parameters: slop: 8720.6, intercept: 438.6) (**B**). The red line represents the background value.

The obtained concentration dependence is given in Figure 8A. The detection limit was 0.86 nM. This value is comparable to the detection limit of the previously studied cis-target (1 nM) [29]. However, this detection limit was worse compared to the previous results and was not sufficient for analytical purposes. Thus, the detection limit of SARS-CoV-2 reached in [31] was equal to several copies per µL. It should be noted that the use of Cas12 does not provide appropriate sensitivity for diagnosis under specific circumstances when an excess of targets is presented. Therefore, CRISPR/Cas12 has been combined with either DNA amplification or special amplification-free approaches with highly sensitive detection based on SERS, electrochemical amplification, digital platforms, enzymes, or nanozymes [32,33,34,35,36].

To realize a more sensitive assay, the CRISPR/Cas12a stage was integrated with pre-amplification of the cis-target DNA using recombinase polymerase amplification. The pre-amplification was performed for 20 min at 39 °C. The mixture after pre-amplification was used for cis-target DNA detection by Cas12 trans-cleavage of the attached ssDNA probe. The efficiency of sequential target DNA pre-amplification and detection using the CRISPR-Cas12 system was demonstrated in several previous studies [11,13,37,38] for different target analytes. This approach combines the high sensitivity of the pre-amplification and the selectivity of the CRISPR-Cas12 system.

We found that the integrated assay protocol lowered the detection limit to several copies (Figure 8B), while a monotonous increase in the fluorescence signal was observed with an increase in the target DNA copies by 4–5 orders of magnitude. The resulting concentration dependence, shown in Figure 8B, corresponded to the linear equation. The detection limit of the proposed combined RPA-CRISPR/Cas12 assay was 4.2 × 10^−17^ M or 50 copies/µL. This detection limit is comparable with previous studies of the Cas12-based fluorescent assays [11,13,37,38,39,40,41] (Table 1).

In general, the CRISPR/Cas12 system requires more additional components (enzymes, RNA, DNA probes, etc.) and more manipulations than PCR or isothermal amplifications. However, the principle of CRISPR/Cas12 functioning provides higher specificity of target DNA recognition. In addition, the proposed CRISPR/Cas12-based biosensor inherits the drawback of each heterogeneous analytical system—its preparation requires the additional assumption of binding reactants due to their loss, inactivation, etc. Nevertheless, the combination of heterogeneous CRISPR/Cas12 with isothermal amplification provides several significant benefits for diagnostic practice: an isothermal regime without the need for a thermal cycler, rapid analysis (the combined system with RPA required 80 min), high sensitivity (detection limit of 50 copies/μL), and high specificity determined by the CRISPR/Cas12 recognition. The approach proposed in the study can be simply transferred to other analytes.

## 4. Conclusions

We compared DNA probes with ds- and ss-parts of different lengths for registering the trans-nuclease activity of Cas12. ssDNA probes were chosen as being more efficient. The initial comparison was undertaken with the fluorescent detection of the released fluorescein residues. Furthermore, three macromolecular reporters were characterized after conducting LFT- and chemiluminescence-based assays. Macromolecules limited the cleavage of the probes, especially short ones, by shielding them from Cas12. Nevertheless, the long ssDNA probes were found to be efficient for heterogeneous CRISPR/Cas12-based assays. A comprehensive comparison of DNA probes with different structures, lengths, and signal molecules showed that the longest tested ssDNA probe (145 nt) proved to be the most efficient one, which is in accordance with it being the least susceptible to steric hindrance.

The finding solutions were applied for the detection of the N-gene of SARS-CoV-2 utilizing Cas12 cleavage of the ssDNA probe (poly-dT, 145 nt, fluorescein reporter) attached to the microplate surface. The system demonstrated a limit of fluorescent detection of 0.86 nM. To reach lower sensitivity, a pre-amplification step using recombinase polymerase amplification (RPA) was introduced. This enhancement led to a significant decrease in the detection limit, achieving an impressive sensitivity of 10^−17^ M. This level of sensitivity is crucial in ensuring the accurate and early detection of SARS-CoV-2 infections, even in samples with very low viral loads.

These findings pave the way for efficient and reliable tools for DNA detection. Furthermore, the successful application of a Cas12-based heterogeneous assay in this study opens up opportunities for the detection of other pathogens and the development of diagnostic devices with high sensitivity and specificity.

## Figures and Tables

**Table 1:** Comparison of the parameters of the CRISPR/Cas12-based assays for the detection of SARS-CoV-2.

Assay	Pre-Amplification	Assay Format	LOD, M
DETECTR [11]	RPA	Homogeneous	~ 10^−18^
HOLMES [13,37]	PCR	Homogeneous	~ 10^−17^
CRISPR-materials [39,40]	RPA	Homogeneous	~ 10^−17^
CDetection [41]	RPA	Homogeneous	~ 10^−18^
HOLMESv2 [38]	LAMP	Homogeneous	~ 10^−17^
Proposed in this study	RPA	Heterogeneous	~ 10^−17^

## Data Availability

The data presented in this study are available on request from the corresponding author.

## Supplementary Materials

The following supporting information can be downloaded at: https://www.mdpi.com/article/10.3390/bios13080824/s1. Figure S1: Characterization of GNPs and GNP-IgG conjugate; Figure S2: Scheme of LFT strip; Figure S3: Detection of anti-FAM with LFT strips: scans of the test zones of the strips and their corresponding color intensity values; Figure S4: Electrophoresis of cis-target DNA; Figure S5: Electrophoresis of in vitro transcribed gRNA for IGS recognition; Figure S6: Kinetic fluorescence curves obtained in result of homogeneous hydrolysis of the ROX-dT(15)-BHQ2 DNA probe by gRNA-Cas12a complex (gRNA purity test); Figure S7: The percentage of DNA probe (ssDNA and dsDNA) of different length cleaved after incubation with anti-FAM coated microplate; Figure S8: Fluorescence of cleaved fluorescein residues of DNA probes and comparison of different blocking proteins; Table S1: Sequences of the primers and oligonucleotide probes for DNA constructs used in this study. Reference [42] is cited in the Supplementary Materials. Table S2: ANOVA statistic.
