# Supplementary material for: A Critical Study on DNA Probes Attached to Microplate for CRISPR/Cas12 Trans-Cleavage Activity
Source: Biosensors (Basel). 2023 Aug 17;13(8):824. doi: 10.3390/bios13080824 (PMC10452489; doi:10.3390/bios13080824)
Supplement: Supplementary file 1 [file biosensors-13-00824-s001.zip › biosensors-2487259-supplementary.pdf]

biosensors

MDPI

Supplementary Materials

A Critical Study on DNA Probes Attached to Microplate for CRISPR/Cas12 Trans-Cleavage Activity

Konstantin M. Burkin, Aleksandr V. Ivanov, Anatoly V. Zherdev, Boris B. Dzantiev and Irina V. Safenkova *

A.N. Bach Institute of Biochemistry, Research Centre of Biotechnology of the Russian Academy of Sciences, 119071 Moscow, Russia
* Correspondence: safenkova@inbi.ras.ru; Tel./Fax: +7-495-954-2804

Section 1. Oligonucleotides and gene Fragments Used in This Study

Table S1. Sequences of the primers and oligonucleotide probes for the DNA constructs used in this study.

Name	Sequence 5′-3′	Purpose
M13 F	GTTGTAAAACGACGGCCAGTG	Synthesis of full length IGS activator DNA
M13 R	AGCGGATAACAATTTCACAC	
N-gene F	TGCCACTAAAGCATACAATG	Synthesis of N-gene fragment from SARS-CoV-2
N-gene R	CTTTGAAATTTGGATCTTTGTC	
gRNA DNA F	TTTTTTTTAATACGACTCACTATAGGTAATTTCTA CTAAGTGTAGATAAACGACGGCATGCGAGCGTG	Matrix for transcription of gRNA for IGS recognition
gRNA DNA R	CACGCTCGCATGCCGTCGTTTATCTACACTTAGT AGAAATTACCTATAGTGAGTCGTATTAAAAAAAA	
ROXdT15-BHQ2	ROX-TTTTTTTTTTTTTTT-BHQ2	Probe for trans-cleavage in the solution
eGFP-F-C3PEG-dT15-FAM	FAM-TTTTTTTTTTTTTTT-(C3Spacer)-GCTACCCCGACCACATGAAG	Primers for combined dsDNA-probe assembly using PCR
eGFP R 20 Bio	Bio-CTTCATGTGGTCGGGGTAGC	
eGFP R 40 Bio	Bio-GACTTGAAGAAGTCGTGCTG	
eGFP R 80 Bio	Bio-GAAGATGGTGCGCTCCTGGA	
eGFP R 120 Bio	Bio-TCACCTCGGCGCGGGTCTTG	
eGFP R 160 Bio	Bio-TTCAGCTCGATGCGGTTCAC	
eGFP R 300 Bio	Bio-TCGATGTTGTGGCGGATCTTG	
eGFP R 500 Bio	Bio-TTACTTGTACAGCTCGTCCATGCCG	
eGFP R 1000 Bio	Bio-AAACTTGATTAGGGTGATGGTTCAC	
FAM–eGFP-0–Bio	FAM-TTTTTTTTTTTTTTT-Bio	Probe for trans-cleavage without the ds-part
gRNA–IGS*	GGUAAUUUCUACUAAGUGUAGAUAAACGACGGCAUGCGA GCGUG	For IGS recognition
gRNA–N-gene	UAAUUUCUACUAAGUGUAGAUCCCCCAGCGCUUCA GCGUUC	For N-gene recognition
FAM–dT(n)–Bio	FAM-T(n)-Biotin	ss-polyT-DNA-probes with lengths of n = 10,

		15, 20, 25, 30, 50, 82, 120, and 145 nt
FAM–dC(n)–Bio	FAM-C(n)-Biotin	ss-polyC-DNA-probes with lengths of n = 10, 20, 30, 50, and 80 nt
FAM–dA(n)–Bio	FAM-A(n)-Biotin	ss-polyA-DNA-probes with lengths of n = 10, 30, 50, and 80 nt
Biotin–dsDNA–PEG–BHQ–ssDNA–FAM	BHQ1-TTTTTTTTTTTTTTT-(C3 spacer)-(T-FAM)GCACCCCGACCACATGAAG	For kinetics

* Site of recognition is denoted by the thick underline.

Full-length IGS activator (596 bp)**

ACGTTGTAAAACGACGGCCAGTGAATTGTAATACGACTCACTATAGGGCGAATTGGGCCCGACGTCGCATGC
TCCCGGCCGCCATGGCGGCCGCGGGAATTCGATGAAAGTGTCTCGGGATGCGGGTATATTGAGAGACTCGAC
CGGCACACAAACTCACGCTCGCATGCCGTCGTTTCAAATTATTCAGCTTGTTCCGGATTGTTAAAGAGCAGAT
AACATAAACCTGACTATCTCTAATCAGTTTTAGGTTAGCGTTGACCGTGCCTTTCACCCACCGTCAGTCATATT
GGCGTCCCCTAGGGGATTCGAACCCCTGTTACCGCCGTGAAAGGGCGGTGTCCTGGGCCTCTAGACGAAGGG
GACATCACTTGTCAGCTTCGCAAGACGCTTTTGACTCTTTCTTATCATCAGACAATCTGTGTGGACACCACGCA
GGCACTTCAAATCACTAGTGAATTCGCGGCCGCCTGCAGGTCGACCATATGGGAGAGCTCCCAACGCGTTG
GATGCATAGCTTGAGTATTCTATAGTGTCACCTAAATAGCTTGGCGTAATCATGGTCATAGCTGTTTCCTGTGT
GAAATTGTTATCCGCT

IGS region of *D. solani* is shown as the light-grey highlighted area. Double underline denotes PAM motive (5′-TTTA-3′). Thick underline denotes gRNA recognition site (along complementary strand in this activator DNA).

Nucleocapsid (N) gene fragment from SARS-CoV-2 (254 bp)**

5′-3′

TGCCACTAAAGCATACAATGTAACACAAGCTTTCGGCAGACGTGGTCCAGAACAAACCCAAGGAAATTTTGG
GGACCAGGAACTAATCAGACAAGGAACTGATTACAAACATTGGCCGCAAATTGCACAATTTGCCCCCAGCG
CTTCAGCGTTCTTCGGAATGTCGCGCATTGGCATGGAAGTCACACCTTCGGGAACGTGGTTGACCTACACAGG
TGCCATCAAATTGGATGACAAAGATCCAAATTTCAAAG

** Sequences of the gene fragments corresponded to the double strand fragments. To simplify, coding strands (5′->3′) are shown only.

Section 2. Preparation of Reagents for Lateral Flow Test Strips and Test Strips Assembling

2.1. Preparation of Conjugate of Gold Nanoparticles (GNPs) with Anti-Mouse-IgG (GAMI)

GNPs were synthesized according to the modified Frens method by reducing $HAuCl_4$ with sodium citrate [42]. The GNPs were characterized with DLS and UV−Vis spectra (**Figure S1**). The GNP solution with 1.0 absorbance units at 520 nM (OD_{520}) was adjusted to a pH of 9.0. Then, GAMI was added to the GNP solution to a final concentration of 10

μg/mL and stirred for 1 h at 20 °C. The surface of the conjugates was blocked by adding BSA to a final concentration of 0.25% (m/v), followed by stirring for 10 min. GNP–IgG conjugates were separated from unbound IgG via centrifugation at 14,000 g for 30 min and successive removal of the supernatant. GNP–IgG conjugates were stored at 4 °C in a 0.25 mM potassium phosphate buffer, a pH of 7.4 with 100 mM of NaCl, 1% BSA, 1% sucrose, 0.025% Tween-20, and 0.1% NaN_3 (ttBSA).

The spectra of the GNPs and their conjugate with GAMI were recorded using a Libra S80 spectrophotometer (Biochrom, UK). Hydrodynamic diameters of GNPs and their conjugate with GAMI were measured using a Zetasizer Nano ZSP (Malvern Panalytical, Malvern, UK), which features a 4 mW He–Ne laser (633 nm). A total of 2 μL of the suspensions were diluted in 200 μL of NEB2.1 buffer (NEB) and inserted into a disposable solvent resistant micro cuvette (ZEN0040; Malvern Panalytical, Malvern, UK). Experimental measurements based on noninvasive backscatter technology were taken with a fixed 173° scattering angle at 25 °C. The experiments were initiated after the sample reached thermal equilibrium. Each sample was measured at least four times, and each measurement consisted of 50 acquisitions. The data were collected and analyzed with Zetasizer Software ver. 8.00. The results are presented in **Figure S1**.

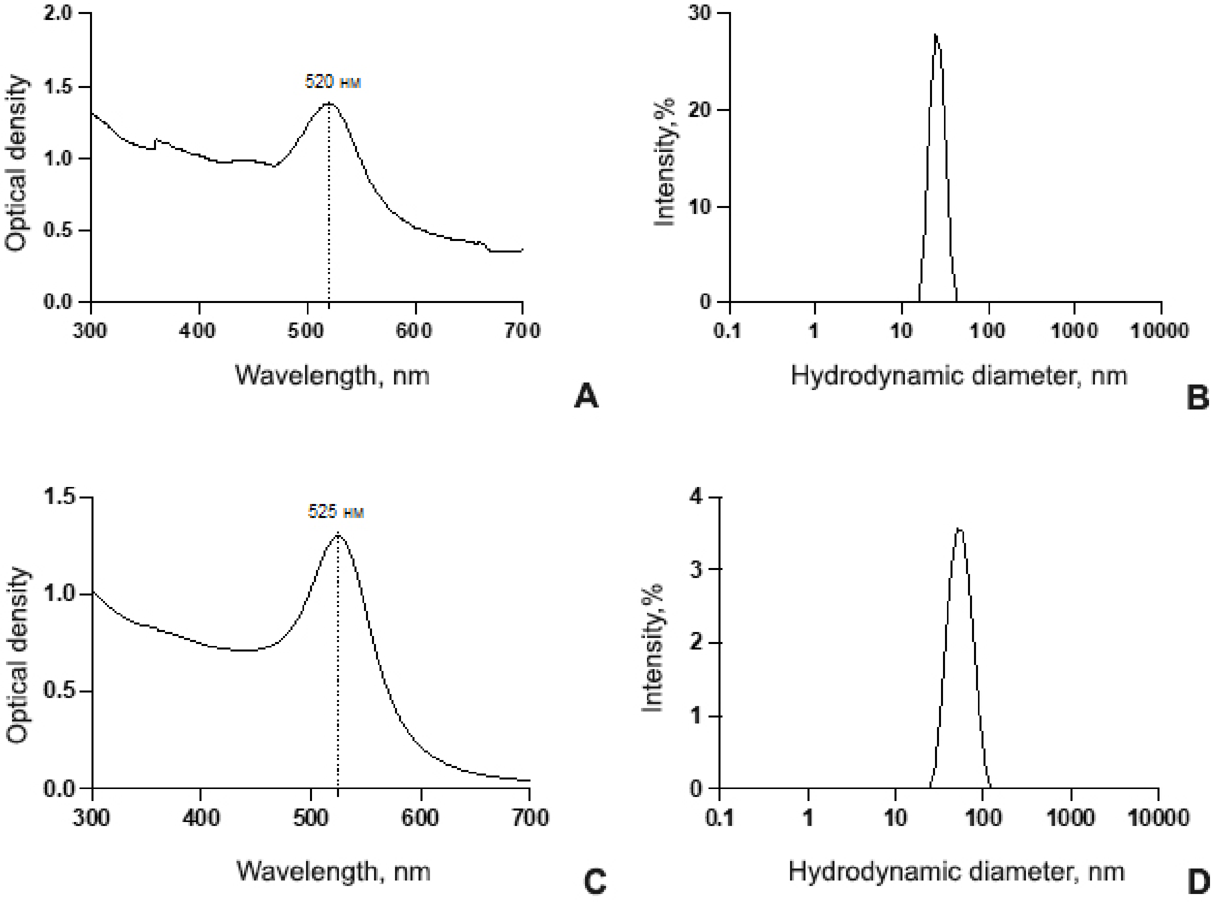

Figure S1. Characterization of the GNPs (**A**, **B**) and GNP–IgG conjugate (**C**, **D**). UV−Vis spectra are presented in (**A**, **C**); distributions of the hydrodynamic diameters are presented in (**B**, **D**).

2.2. Preparation of Lateral Flow Test (LFT) Strips and Their Characterization

The LFT strips were assembled using plastic supports with a UniSart CN-95 nitrocellulose membrane (Sartorius, Göttingen, Germany); other membranes like the PT-R5 glass fiber membrane (conjugate pad), GFB-R4 sample pad, and AP045 absorbent pad were purchased from Advanced Microdevices (Ambala Cantt, Haryana, India) according to the

scheme in **Figure S2**. Anti-mouse-IgG (GAMI) were dispensed on the test zone, and non-specific monoclonal mouse antibodies (NT-MAb) were dispensed on the control zone using an IsoFlow Dispenser (Imagene Technology, St. Lebanon, NH, USA) with a dispersion rate of 0.15 µL per mm of CN 95 nitrocellulose membrane; all components used 1 mg/mL in a 50 mM potassium phosphate buffer, with a pH of 7.4 with 100 mM of NaCl, 5% glycerol, and 0.03% NaN_3. The synthesized conjugate of GNP–GAMI (OD_{520} = 3.5) was dispensed manually at 1.6 µL per 4 mm^2 of conjugate pad. The multimembrane composites were dried after dispensing at 20 °C overnight and then cut into 4-mm strips using an automatic guillotine ZQ2002 (Shanghai Kinbio Tech, Shanghai.

The obtained LFT strips were tested to detect anti-FAM at concentrations from 500 to 0.5 ng/mL. The test strip was dipped in 100 µL of the antibody solution. After drying up, the test strip was scanned by a Canon Scanner (Tokyo, Japan); scans were further analyzed. **Figure S3** shows a scan of the test zones of the LFT strips and their corresponding color intensity values. The obtained results showed that the test strips detected anti-FAM in the range from 5 to 500 ng/ml, which is a sufficient range for the detection of anti-FAM in the CRISPR/Cas system after trans-nuclease reaction.

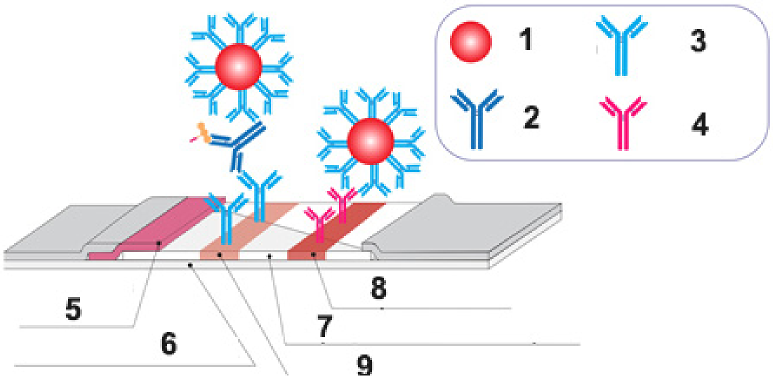

Figure S2. Scheme of the LFT strip. The numbers represent GNP (1), anti-FAM (**2**), GAMI (**3**), NT-MAb (**4**), conjugate pad (**5**), plastic support (**6**), nitrocellulose membrane (**7**), control zone (**8**), test zone (**9**). Direction of fluid movement from left to right.

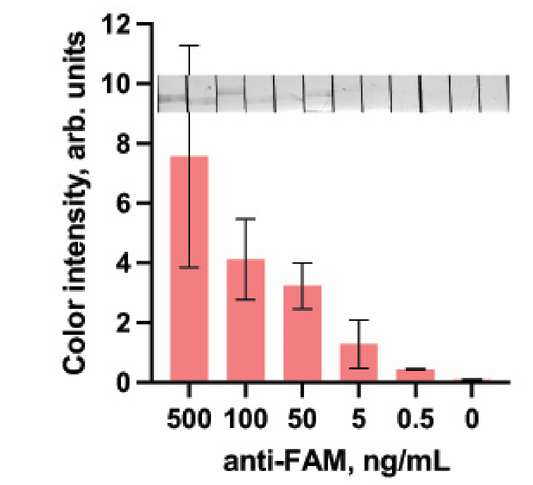

Figure S3. Detection of anti-FAM with the LFT strips: scans of the test zones of the strips and their corresponding color intensity values. Anti-FAM at a concentration of 500 to 0.5 ng/mL, 2 repetitions for each concentration.

Section 3. Characterization of the Obtained DNA and RNA Components for Heterogeneous CRISPR/Cas12 Trans-Cleavage

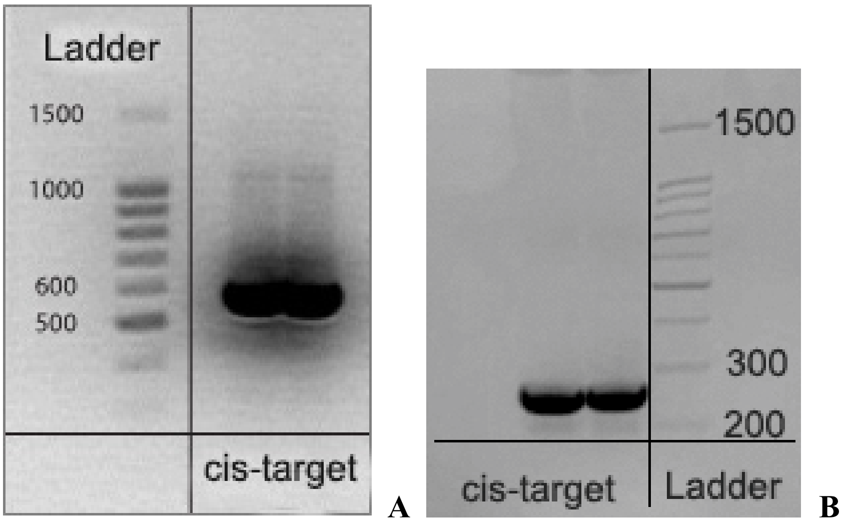

Figure S4. Electrophoresis of cis-target DNA. (**A**) *IGS Dickeya solani*, 596 bp, (**B**) *N-gene SARS-CoV-2*, 254 bp.

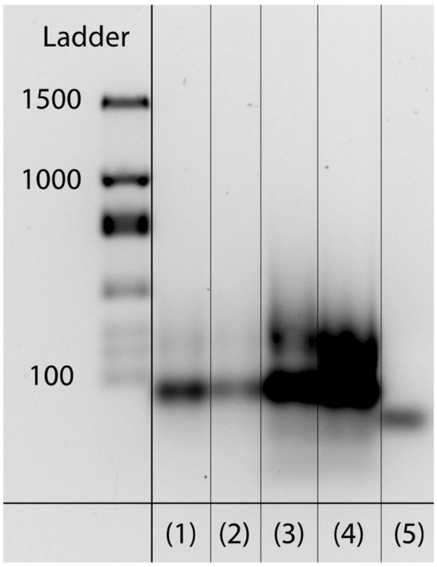

Figure S5. Electrophoresis of in vitro transcribed gRNA for IGS recognition. (**1**) gRNA 2x concentration; (**2**) gRNA; (**3**) gRNA after DNAse; (**4**) gRNA after transcription; (**5**) 21 nt primer.

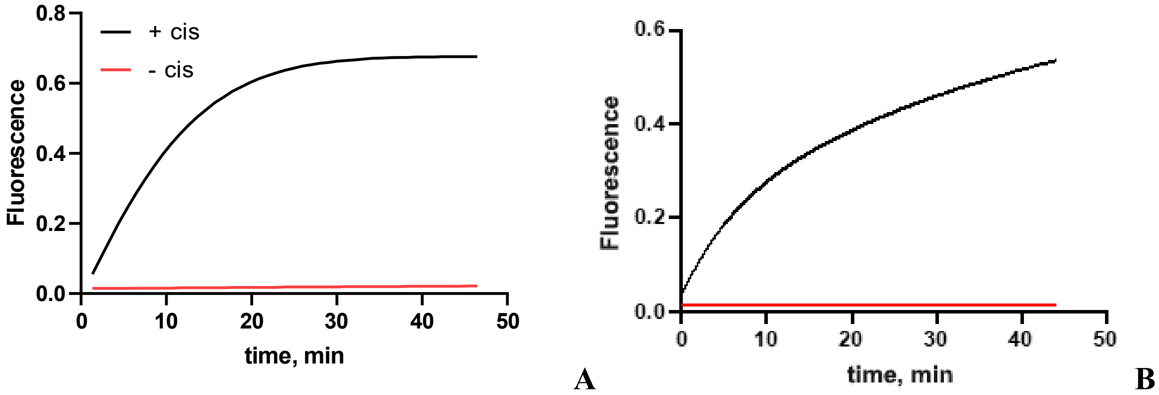

Figure S6. Kinetic fluorescence curves obtained as a result of the homogeneous hydrolysis of the ROX-dT(15)-BHQ2 DNA probe by the gRNA–Cas12a complex (gRNA purity test). Black curves

correspond to Cas12a activated by cis-target DNA, and red curves to the background responses in the absence of cis-target DNA. (**A**) gRNA for IGS, (**B**) gRNA for the N-gene. The curves are based on the results of measuring the fluorescence every 30 s.

Section 4. Optimization of Conditions for Heterogeneous CRISPR/Cas12 Trans-Cleavage

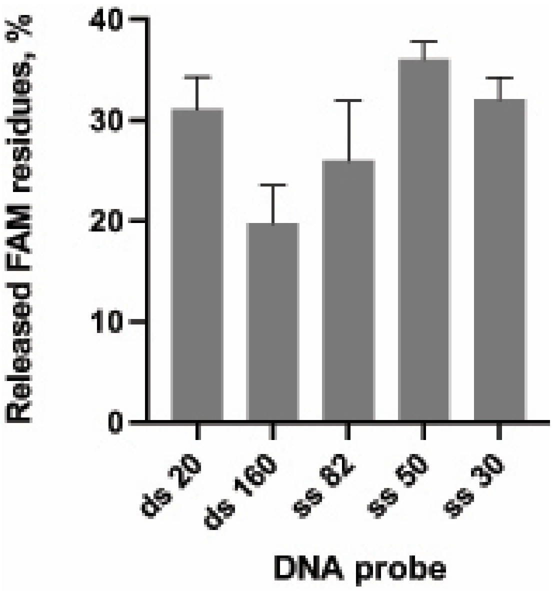

Figure S7. The percentage of DNA probe (ssDNA and dsDNA) of different lengths cleaved after incubation with the anti-FAM coated microplate. The DNA probes were used in a 50 nM concentration.

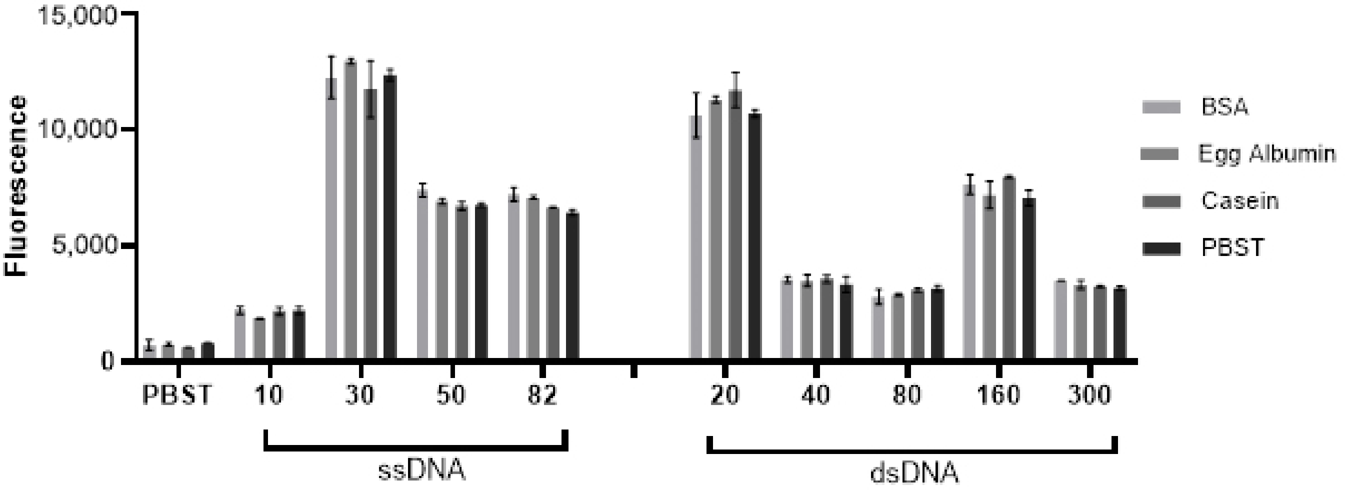

Figure S8. Fluorescence of the cleaved fluorescein residues of the DNA probes and a comparison of the different blocking proteins.
